# Surgical management of pseudoaneurysm after subclavian artery-to-descending aorta bypass in an adult patient with aortic coarctation

**DOI:** 10.1186/s44215-024-00154-6

**Published:** 2024-05-02

**Authors:** Kyohei Hatori, Jun Mohara, Miyuki Murata, Tetsuya Koyano

**Affiliations:** Department of Cardiovascular Surgery, NHO Takasaki General Medical Center, 36 Takamatu, Takasaki, Gunma 372-0829 Japan

**Keywords:** Coarctation of the aorta, Aortic coarctation, Adult, Pseudoaneurysm, Frozen elephant trunk

## Abstract

**Background:**

Coarctation of the aorta is a relatively common congenital heart defect. Various operative techniques have been proposed for aortic coarctation repair, which are tailored to individual patient circumstances. However, regardless of the chosen surgical approach, some patients may develop late thoracic pseudoaneurysms. Repeated surgery with a left thoracotomy increases the risk of lung injury and bleeding due to adhesions and rich collateral blood circulation.

**Case presentation:**

The patient underwent subclavian artery descending aortic bypass surgery for coarctation of the aorta at the age of 22 years and developed a pseudoaneurysm 30 years after bypass surgery. From the available surgical options for this condition, we selected total arch replacement using a frozen elephant trunk. The outcomes were excellent.

**Conclusions:**

Depending on each patient’s circumstances, revision surgery using the frozen elephant trunk technique can be a viable option.

## Background

Coarctation of the aorta (CoA) is a relatively common congenital heart defect, affecting 5–8% of patients with congenital heart disease [1]. Several operative techniques have been proposed for CoA repair depending on the individual patient’s circumstances. However, regardless of the chosen surgery, some patients develop late thoracic pseudoaneurysms [2]. From the available surgical options for this condition, we selected a total arch replacement with a frozen elephant trunk (FET) via median sternotomy. Here, we report our experience and discuss the characteristics of adult CoA and the benefits of FET.

## Case presentation

**Fig. 1:**
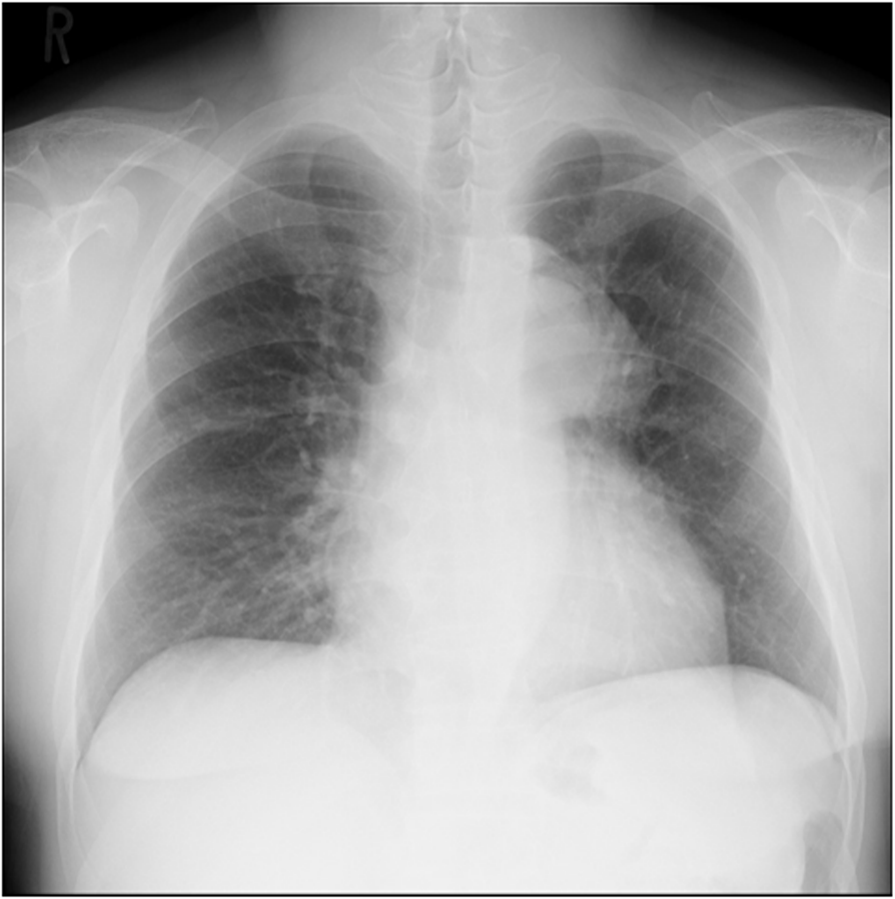
Preoperative chest radiography showing enlargement of the superior mediastinum

**Fig. 2:**
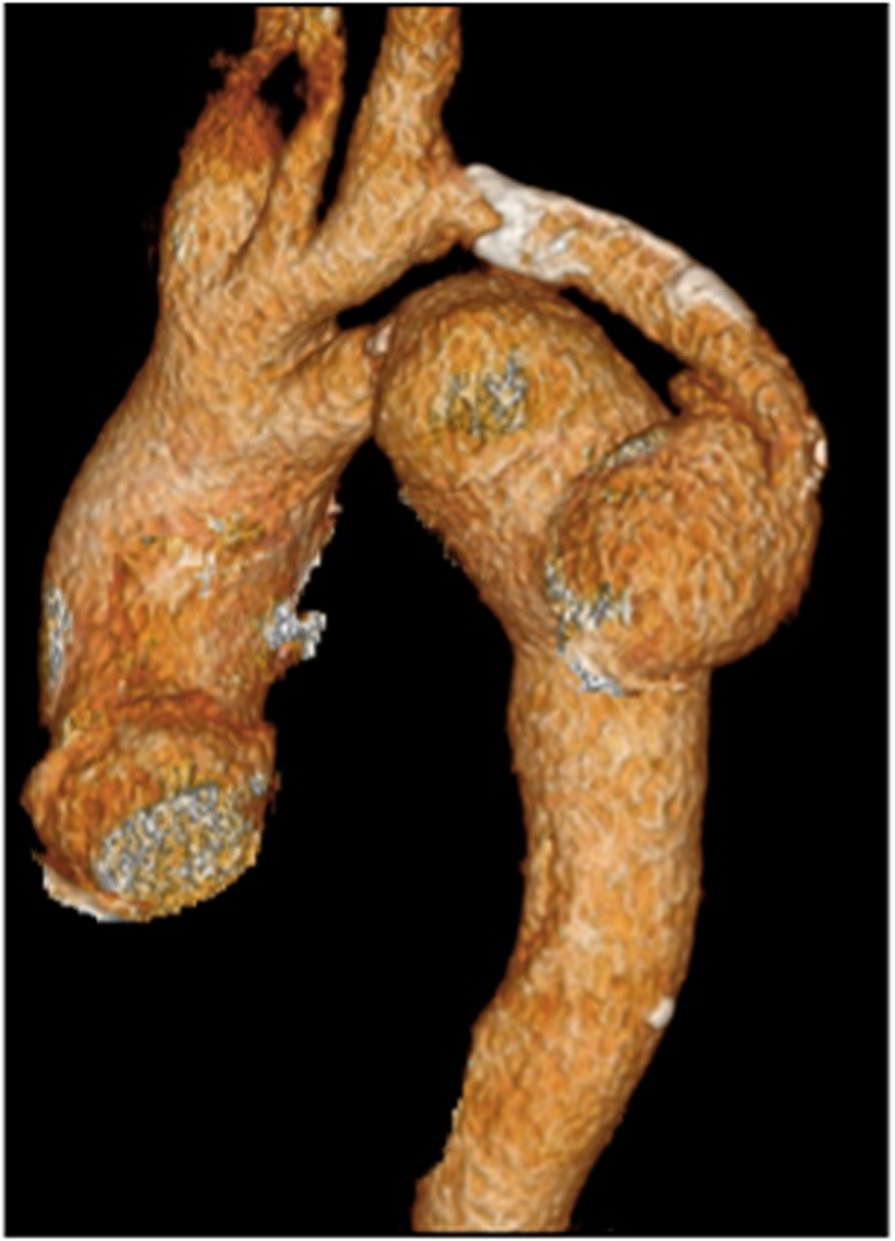
Three-dimensional computed tomography. Preoperative view. A pseudoaneurysm was formed at the distal anastomosis of the bypass graft

**Fig. 3:**
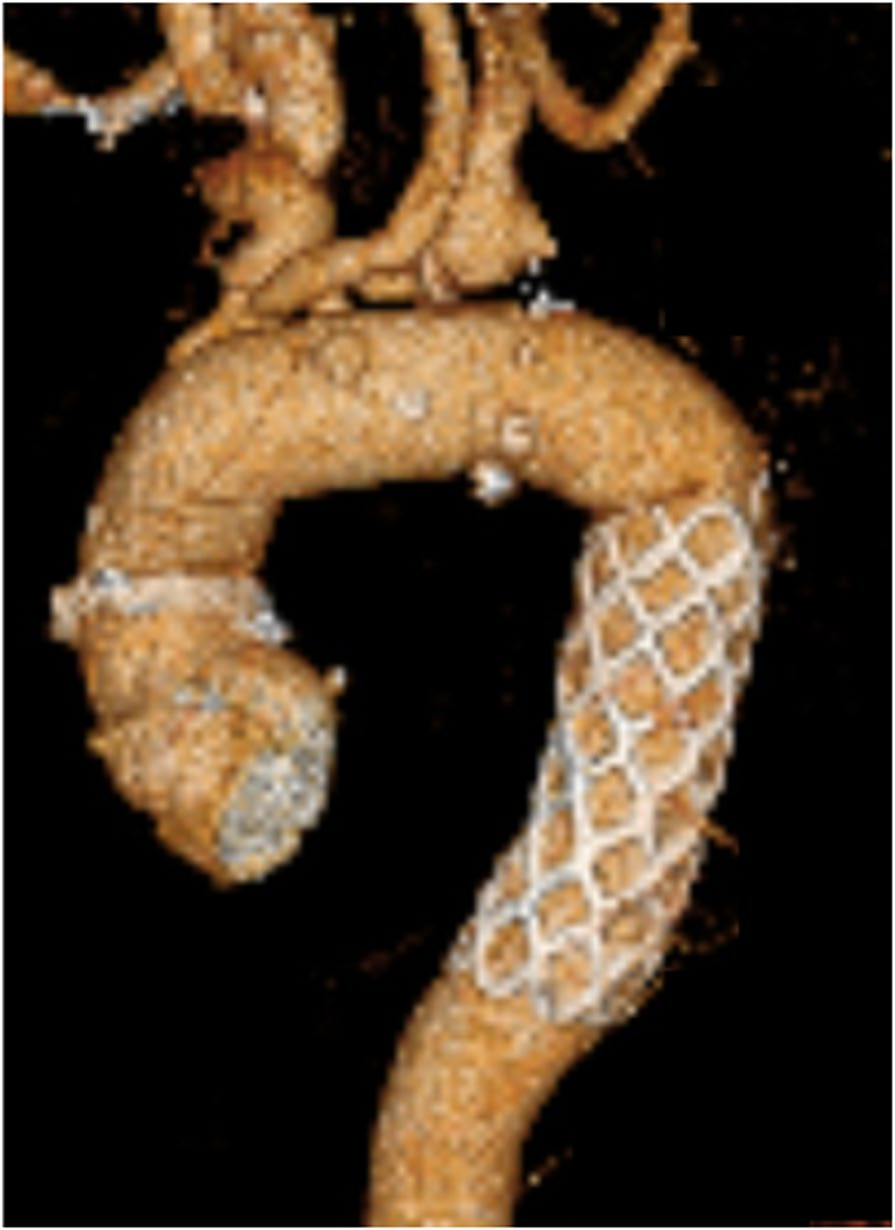
Three-dimensional computed tomography. Postoperative view. The coarcted aorta was resected, and the frozen elephant trunk was inserted into the descending aorta

A 52-year-old man, who had previously undergone surgery for CoA at the age of 22, fell from a height of 5 m. Following this incident, the patient continued to experience back pain and bloody sputum. Two months later, the patient visited a medical institution, where a computed tomography (CT) scan revealed a descending aortic pseudoaneurysm. He was subsequently referred to our hospital for treatment. The patient had a history of hypertension and had been on medication for an extended period. His brachial blood pressure was 155/96 mmHg, whereas his ankle blood pressure was 145/82 mmHg. Chest radiography indicated enlargement of the superior mediastinum (Fig. 1). Preoperative CT revealed a 32-mm-diameter aortic arch, with the aorta coarcted distal to the ductus arteriosus. In addition, it was noted that the subclavian artery had been bypassed to the descending aorta using a 14-mm-diameter prosthetic graft (Fig. 2). Pseudoaneurysm developed at the distal anastomosis of the prosthetic graft and expanded to 46 mm. The diameter of the descending aorta was 27 mm. Although persistent hemoptysis and back pain could have been caused by chest and back contusions and pulmonary contusions, we could not entirely rule out the possibility that the pseudoaneurysm was on the verge of rupture due to trauma. We opted for a one-stage surgical intervention. Total arch replacement and FET were conducted as follows. Under general anesthesia, a median sternotomy was performed, and the patient was placed on cardiopulmonary bypass support using the ascending aorta and superior and inferior vena cava. A left ventricular venting tube was inserted through the right upper pulmonary vein. During whole-body cooling, the left subclavian artery and the proximal side of the previous vascular graft were exposed. This graft was ligated at the proximal end. After clamping the ascending aorta, myocardial protection was ensured by antegrade intermittent infusion of a cold blood cardioplegic solution. With a rectal temperature of 27 °C, circulatory arrest was induced. We opened the aortic arch and performed selective cerebral perfusion using balloon catheters. The aortic arch was incised longitudinally to the tip of the coarcted aorta, and the descending aorta was transected at the distal end of the coarcted area. We introduced a J graft Frozenix® (Japan LifeLine, Tokyo) into the descending aorta to monitor transesophageal echocardiography. The distal end of the FET was positioned at the level of the 9th thoracic vertebra. It was then anastomosed using a Triplex® four-branched graft (Terumo, Tokyo, Japan). The Frozenix® graft had a diameter of 29 mm and a length of 90 mm, whereas the Triplex® graft had a diameter of 28 mm. The left subclavian artery and the prosthetic graft branch were anastomosed. A vascular graft was clamped proximally, antegrade systemic circulation was restarted through the side branch of the prosthesis, and rewarming of the whole body was initiated. Subsequently, anastomoses were constructed in the proximal root, left carotid artery, and right brachiocephalic artery. The operative time was 474 min, cardiac arrest time was 132 min, systemic circulation arrest time was 73 min, and selective cerebral perfusion time was 143 min. The patient developed hoarseness due to a left recurrent laryngeal nerve injury. However, apart from this complication, the patient’s recovery was uneventful, and he was discharged from the hospital 25 days after the surgery. Recurrence of CoA or pseudoaneurysm was not shown on 3D-CT 2 years after surgery (Fig. 3).

## Discussion

CoA is a relatively common congenital heart defect that affects 5–8% of individuals with congenital heart disease. Fortunately, almost all cases of CoA are now identified and treated in early childhood. However, there are rare instances where CoA is diagnosed in adulthood, presenting with symptoms such as hypertension, leg fatigue, headache, or heart failure. Although treatment for pediatric CoA is well established, managing CoA in adults poses significant challenges. The vessels are more friable, and the intercostal arteries are larger and more prone to damage, making dissection potentially hazardous [3]. Various operative techniques have been proposed for CoA repair in adults, depending on the individual patient’s circumstances. Extra-anatomical bypass grafting is preferred because it is easier to perform and tension-free [4, 5]. Therefore, it seemed that bypass treatment was initially chosen for our patient.

Some reports have raised concerns about the fragility of the descending aorta in patients with CoA [4, 6]. A proposed theory suggests that coarctation may represent a diffuse aortopathy because it is associated with subsequent aneurysm formation not only at the site of previous repair but also in contiguous and remote segments of the aorta and branch arteries [2]. A comprehensive study evaluating long-term results from 891 patients after open CoA repair (1 to 24 years prior) revealed that 48 (5.4%) developed pseudo-aneurysms at the repair site: 89.6% underwent patch aortoplasty, 8.3% after end-to-end anastomosis, and 2.1% with prosthetic graft replacement [7]. Nowadays, patch aortoplasty is outdated. Therefore, in adult congenital patients, it is important to be mindful of complications arising from past surgical procedures. Given these considerations, the 2018 AHA/ACC guidelines for adult congenital heart disease recommend that regardless of the chosen treatment approach, all patients must undergo regular follow-up and surveillance imaging after CoA repair [8]. This proactive monitoring is crucial for detecting potential complications and ensuring optimal long-term outcomes.

While left thoracotomy with descending aortic replacement and total arch replacement were viable options, we ultimately opted against the left thoracotomy approach for three reasons. First, we recognized that operating on the stenotic segment and the deeper portion of the aortic arch would be challenging. Second, although it was mild, there was a difference in blood pressure between the upper and lower limbs. We thought that the intercostal arteries were well-developed. Therefore, we needed to exercise caution regarding the potentially rich blood flow in the chest wall. Third, reoperation via left thoracotomy has a significant incidence of postoperative complications, with reported mortality and morbidity rates ranging from 0 to 8% and 50%, respectively [9].

The FET technique eliminates the need for surgical replacement of the descending aorta, thus avoiding the need to separate adhesions between the lung and pseudoaneurysm. Idrees et al. reported on 14 patients with aortic aneurysms associated with previous coarctation who underwent hybrid repair using various techniques: elephant trunk with endovascular completion (*n* = 5), frozen elephant trunk (*n* = 8), or antegrade stent grafting (*n* = 1). The results of these hybrid repairs were found to be safe and effective [10]. Notably, the occurrence of stent-graft-induced new entry was quite low because of the internal stent skeleton of the Frozenix® device [11]. Therefore, this procedure is well-suited for treating fragile blood vessels, such as those observed in adult CoA cases.

During surgery, the recurrent laryngeal nerve was visually identified at the level of the aortic arch. However, postoperatively, the patient developed hoarseness because of a left recurrent laryngeal nerve injury. The surgical procedure involved deeper manipulation than standard total arch replacement and FET procedures. While identification was achieved, in retrospect, greater caution should have been exercised during the manipulation of the recurrent nerve and its surrounding tissues.

## Conclusion

We report a case of pseudoaneurysm in the late period after CoA surgery. The initial treatment of CoA in adult patients presents different challenges than those encountered in infants. Therefore, CoA revision surgery involves various situations. Depending on each patient’s circumstances, revision surgery with FET can be a viable option.

## Data Availability

Not applicable.

## Abbreviations

CoA: Coarctation of the aorta
FET: Frozen elephant trunk
CT: Computed tomography
